# “Leave your smartphone out of bed”: quantitative analysis of smartphone use effect on sleep quality

**DOI:** 10.1007/s00779-022-01694-w

**Published:** 2022-11-09

**Authors:** Saba Kheirinejad, Aku Visuri, Denzil Ferreira, Simo Hosio

**Affiliations:** University of Oulu, Oulu, Finland

**Keywords:** Sleep, Smartphone, Application, Wearable sensors

## Abstract

Smartphones have become an integral part of people’s everyday lives. Smartphones are used across all household locations, including in the bed at night. Smartphone screens and other displays emit blue light, and exposure to blue light can affect one’s sleep quality. Thus, smartphone use prior to bedtime could disrupt the quality of one’s sleep, but research lacks quantitative studies on how smartphone use can influence sleep. This study combines smartphone application use data from 75 participants with sleep data collected by a wearable ring. On average, the participants used their smartphones in bed for 322.8 s (5 min and 22.8 s), with an IQR of 43.7–456. Participants spent an average of 42% of their time in bed using their smartphones (IQR of 5.87–55.5%). Our findings indicate that smartphone use in bed has significant adverse effects on sleep latency, awake time, average heart rate, and HR variability. We also find that smartphone use does not decrease sleep quality when used outside of bed. Our results indicate that intense smartphone use alone does not negatively affect well-being. Since all smartphone users do not use their phones in the same way, extending the investigation to different smartphone use types might yield more information than general smartphone use. In conclusion, this paper presents the first investigation of the association between smartphone application use logs and *detailed* sleep metrics. Our work also validates previous research results and highlights emerging future work.

## Introduction

Technology has become an integral part of people’s lives, and the most widely adopted technology is the smartphone. At the beginning of the year 2021, the number of smartphone users in the world is 3.8 billion, which shows that 48.53% of the world’s population owns a smartphone.^1^ Smartphones are devices beyond mobile phones that were just simple devices to communicate. Nowadays, one can go out without their wallet or bank card, as most required functions can be done using one’s smartphone. Despite all the positive aspects of smartphones, their overuse, especially at night, has increased concerns about their potential adverse effects on mental health and sleep, especially in youth [1, 2]. Since smartphones are easy to carry, people carry them with themselves everywhere. They take their smartphone to bed and entertain themselves for hours.

Human beings spend almost one-third time of the day sleeping. Sleep is one of the inevitable daily life activities which plays an important role in the health, well-being, and quality of daily life. Sleep influences our brain functionality through the ability to learn, memorize, and make logical decisions [3]. Sleeping well can boost the immune system [4]. Sleep deprivation has significant effects on performance and concentration [5]. Some factors affect sleep quality, such as environmental factors [6–8], psychological factors [9, 10], and biological factors [11].

Intense use of media or every device with screens that emit light such as TV, computer, tablet, and smartphone could lead to poor sleep quality [12–14]. The light emitted from the screens of mentioned devices is intense in the blue part of the visible spectrum of light. That type of light strongly stimulates retinal ganglion cells containing melanopsin. Stimulation of those cells causes secretion inhibition of melatonin and decreases sleepiness. Thus, using smartphones, especially during the night, disrupts the normal sleep-wake cycle, sleep delay, and melatonin secretion inhibition [15]. Some findings show that intense smartphone use alone does not negatively affect well-being. Since all smartphone users do not use their phones the same way, investigating smartphone use types might yield more information than general smartphone use. Thus, investigating smartphone use in more detail can lead people to use the technology’s advantages well and prevent the disadvantages [16]. Most studies specifically investigated the impact of smartphone use on sleep considered the social media category. They indicate a practically and statistically significant relationship between social media use and sleep patterns, particularly late sleep onset [17–20]. Most studies have investigated the effect of smartphone use at times that are close to bedtime [21, 22]. To the best of our knowledge, no study has investigated the impact of using different smartphone applications at different times of the day on sleep quality, nor how a specific type requires different cognitive functions of smartphone use (“active” or “passive”) influences sleep quality.

This paper presents the findings of a 4-month study designed to investigate how smartphone use influences sleep quality. We capture smartphone use factors through the participant’s smartphone and sleep quality collected through a wearable ring sensor. We offer a quantitative analysis of both datasets and reveal how previously known factors influencing sleep quality can be measured in such datasets. Additionally, we introduce new potential research topics for future technology use and how it influences sleep quality. Our contributions are as follows:

- We show how smartphone use *in bed* can decrease sleep quality, and primarily using smartphone *before bedtime* can have a positive effect on sleep quality.
- Our results illustrate that *not all *smartphone application use affects one’s sleep quality in the same way.
- We show how smartphone use in bed affects *sleep metrics* such as sleep latency, night-time heart rate, RMSSD, deep sleep, light sleep, REM, awake time, and restlessness and offer quantified information of said effect(s). It is worth noting that wearable proprietary algorithms determine these metrics.
- Finally, we highlight potential future research agendas by distinguishing applications that require *typing* and hypothesize how the increased requirement for cognitive function in these applications can influence sleep quality and recovery.

## Related work

### Sleep and its constituents

Sleep is an essential factor in health [23], and it affects various aspects of our daily life quality [24] and our physiology and behavior [25]. Sleeping well boosts the immune system [4]. It also influences brain functionality, including learning, memorizing, and making logical decisions [3]. Many factors affect the quality of sleep, e.g., environmental factors [26] such as light, noise, temperature [27], air quality [6], seasonal changes, exposure to smoking in the sleeping room, a feature of bed and pillow, and moisture of the room; psychological factors such as depression, excitement, stress, anxiety, and fear; biological factors such as pain, drug use, alcohol use, consuming more tea and coffee, feeling of hunger, excessive eating, and fatigue; and social factors such as having a problem with family and friend, financial distress, having trouble at school or work, and loneliness [11]. Daily activities like physical exercise [28] also influence how we sleep [29]. Sleep deprivation has significant effects on performance and concentration [5]. Studies showed that poorer sleepers reported significantly more pain (back pain, headache, etc.) [30, 31].

Sleep hygiene is a set of behaviors that enhance sleep quality and quantity [32]. Different factors can affect sleep hygiene, such as nutritional factors or diet, that might reduce sleep duration and quality [33]. Dependent on the individual’s tolerance, caffeine might impair sleep. Alcohol intake before sleep can be adverse to sleep quality and quantity. Cigarette smoking may diminish memory and negatively affects sleep quality [34]. Hyper-hydration and waking during the night to urinate also lead to sleep disturbance [35]. High or low air temperatures could significantly impair sleep quality [36]. Sleep seems to be sensitive to ambient noise since the sensory functions of the sleeper process external stimuli [37–40]. Studies indicate that short naps and moderate exercise, such as walking in the evening, play a significant role in enhancing the sleep quality in older people [41]. Exposure to bright light, caffeine consumption, or face washing after a short nap can reduce mid-afternoon sleepiness [42]. The living place is another factor that can improve sleep; for instance, green space exposure can enhance sleep quality and quantity [43]. Increasing shreds of evidence show that individuals who live in greener areas have better sleep quality [44, 45]. There are some self-help methods to enhance sleep. Clinical studies indicate that music can promote human emotions and treatment results. Accordingly, music is one of the most-used self-help methods to improve sleep quality [46]. Self-relaxation could be effective on cognitive functions and sleep quality in the elders [47]. Some findings support the evidence that smartphone technologies can effectively enhance sleep quality and solve sleep disorders [48, 49].

There are several methods to measure sleep quality, including clinical modalities such as polysomnography [50, 51], multiple sleep latency tests (MSLT), maintenance of wakefulness test (MWT), and home sleep apnea testing (HSAT); consumer technology like smartphone applications [52, 53], wearable trackers [54, 55], and non-wearable tracker [56]; and crowdsourcing sleep research or self-assessment methods like sleep questionnaires or sleep diaries [57, 58]. Most wearable trackers use accelerometer sensors, heat flux sensors, and optical blood-flow sensors [59]. There is no perfect sleep assessment method; all methods have advantages and disadvantages. The few scientific validation studies that have compared smartphone applications against polysomnography report that they are not useful as a tool for sleep estimation [60] and they are still not accurate enough to be used as clinical tools [58, 61].

**Fig. 1:**
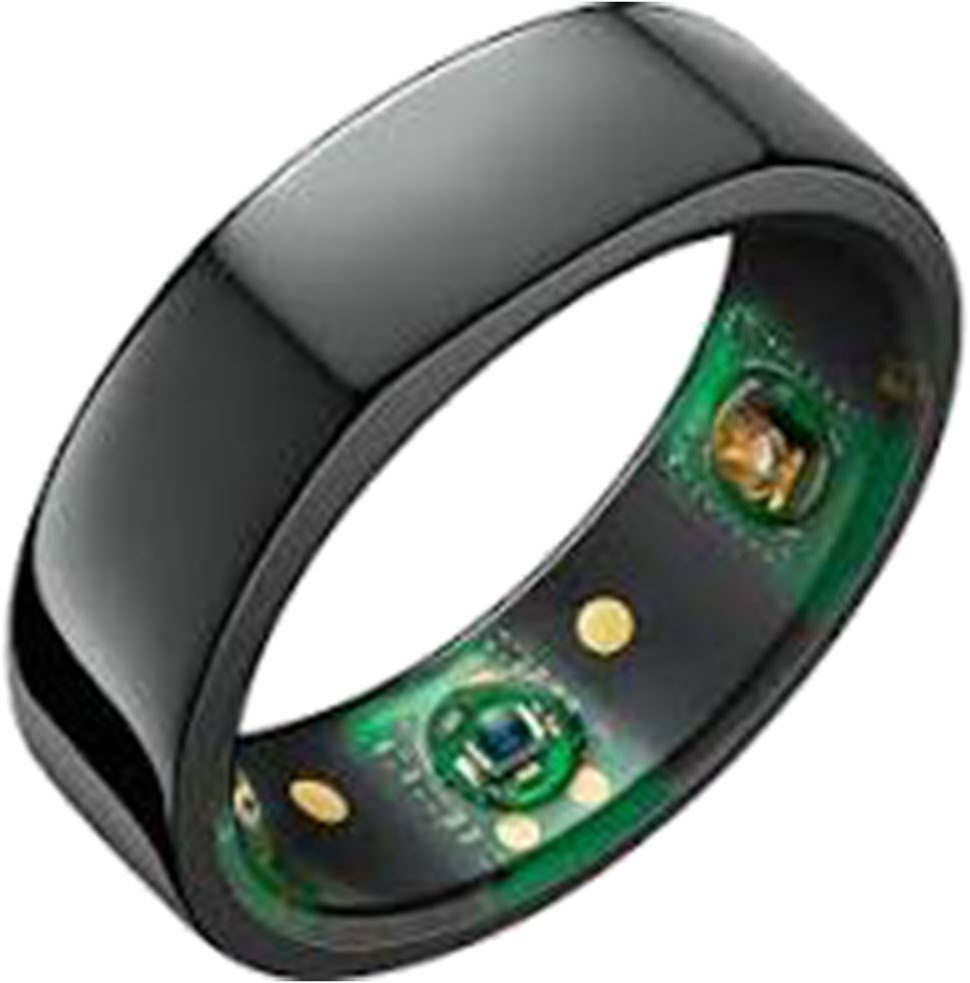
The Oura sleep tracking ring. The sensors — infrared photoplethysmography (PPG) for heart rate, negative temperature coefficient (NTC) for temperature, and 3D accelerometer for movement — are placed on the inner surface of the ring

Some clinicians are skeptical of the accuracy and use of sleep information offered by consumer devices [55, 57]. However, the Oura ring is a state-of-the-art wearable sleep tracker and a promising tool for conducting large-scale studies and health management [62]. Validation studies indicated generally good accuracy of sleep-wake detection for the Oura ring, with small absolute error for total sleep time estimation compared to polysomnography (PSG; 87.8% of nights within 30-min error) [63], ambulatory electroencephalography (7.39% mean absolute percentage error) [64], and 94% accuracy for 2-stage detection (sleep, wake) compared to polysomnography for a simple accelerometer-based model and 96% for a full model using machine learning algorithm [62]. It could accurately measure nocturnal heart rate (HR) and average heart rate variability (RMSSD) in both the 5-min and average-per-night tests compared to an electrocardiogram device [65]. It also outperforms other wearable devices such as smartwatches [66]. The Oura ring combines advanced sensor technology and a minimal design with an easy-to-use mobile app to deliver precise, personalized health insights straight from the most reliable source: your body. The Oura ring has three sensors: a 3D accelerometer for movement, infrared photoplethysmography sensors (PPG) for heart rate and respiration, and a negative temperature coefficient (NTC) sensor for body temperature [67] (Fig. 1).

### Smartphones and sleep

Presently, smartphones are integrated into different aspects of daily life. They are accommodating for performing tasks or communicating with friends. However, the overuse of smartphones and the Internet, especially at night, has increased concerns about their potential mental health and sleep effects, especially in youth [68, 69]. In addition to mental and behavioral health problems, smartphone overuse can lead to physical health problems such as blurred vision and pain in the hand, back, neck, and head [70]. It has been observed that frequency and headache duration in migraine patients increases with smartphone use. Therefore, smartphone overuse in migraine patients leads to sleep quality reduction and daytime sleepiness increment [71]. Most studies that proposed preliminary insights into behavioral patterns between smartphone use and sleep quality used self-reported questionnaires [72, 73].

Studies revealed that smartphone addiction [1, 74–78] or intensive use [73, 79–81] is a risk factor for poor sleep quality. It can lead to sleep latency [82]. Intense use of media or every device with screens that emit light such as a TV, computer, tablet, and smartphone could lead to poor sleep quality [12–14]. The light emitted from the screens of smartphones is strong in the blue part of the visible spectrum of light. That type of light strongly stimulates retinal ganglion cells containing melanopsin. Stimulation of those cells causes secretion inhibition of melatonin and decreases sleepiness. Therefore, using a smartphone during the night causes a delay in melatonin secretion and disrupts the sleep-wake cycle [15]. According to this, studies show that the distance of the smartphone from the eyes affects sleep quality [83]. Since smartphones are small enough to carry in bed, smartphone use in bed is swiftly becoming commonplace. Therefore, it is reasonable to see adverse impacts on sleep quality and daytime functioning [22, 84, 85]. Thus, to improve health and wellness in adolescents, individuals should set boundaries on smartphone use, especially at bedtime [86]. Given that people use smartphones in bed, there is a bidirectional relation between the duration of smartphone use and various sleep disturbances [87]. Findings show that screen time is associated with poor sleep. However, they could not exactly determine that which one is the cause. Poor sleep leads to screen time increment or screen time increment leads to poor sleep [88].

Studies indicate that intense smartphone use alone does not lead to negative well-being [16]. Since all smartphone users do not use their phones the same way, investigating smartphone use types might yield more information than general smartphone use. Therefore, investigating smartphone use in more detail can lead people to use the advantages of the technology well and prevent the disadvantages. The only applications that have been specifically investigated are social media such as Facebook, Instagram, and Twitter. At the beginning of the year 2021, the number of smartphone users in the world is 3.8 billion, and there are 3.78 billion social media users worldwide.^2^ Social media is regarded as a “double-edged sword.” They have broken the geographic and time zone boundaries, keep the world “always connected” [89], and enable people to express their thoughts and feelings and receive social support [90–93]. However, they have negative consequences, such as poorer sleep quality and worse mental health [94, 95], e.g., “the Facebook depression phenomenon,” which is due to the social comparisons [19]. Using social media, especially at night, is associated with higher anxiety and depression levels, lower self-esteem, and poorer sleep quality [20]. Scott et al. indicated a practically and statistically significant relationship between social media use and sleep patterns, particularly late sleep onset [17]. Social media use before going to bed is independently related to disturbed sleep among young adults [21]. Bhat et al. showed that using social media in bed causes sleep and mode dysfunction in adults [22]. Levenson et al. showed that participants with higher social media use per day and per week had significantly greater sleep disturbance [96].

## Study design

This work presents a 4-month-long experiment intended to measure how smartphones influence sleep quality. We collect the following metrics during the experiment to gain more insight into this relationship. Firstly, we use a wearable sleep-tracking ring to collect sleep quality, quantity, and individual sleep metrics during the whole study period. Second, we collect smartphone use behavior (typing and application use) from the participants’ personal devices during the study period. The following section will describe details of our overall study plan and descriptions and functionalities of the technologies used to collect our dataset. Based on related work, we formulated the following research questions and our initial hypotheses on them:

- **RQ1**: Does more smartphone use during bedtime or throughout the day generally translate into worse or better sleep quality?
  **Hypothesis (H1):** More smartphone use, especially *overuse* of smartphones, can lead to a decline in sleep quality.
- **RQ2**: Does the use of specific application types correspond with worse or better sleep quality?
  **H2:** According to generally accepted theories [17, 97, 98], we should see differences for some application types, e.g., social media applications causing worse sleep quality
- **RQ3**: Does the use of applications requiring active involvement in the form of typing have an impact on sleep quality?
  **H3:** More cognitively demanding applications used, especially late at night, would likely cause a decline in sleep quality or quantity.
- **RQ4:** How do smartphone use and typing activities influence detailed aspects of sleep (e.g., sleep phases, sleep latency.)
  **Hypothesis:** Without prior research focus on these *detailed sleep metrics*, we do not offer any initial hypothesis.

### Study plan and recruitment

**Fig. 2:**
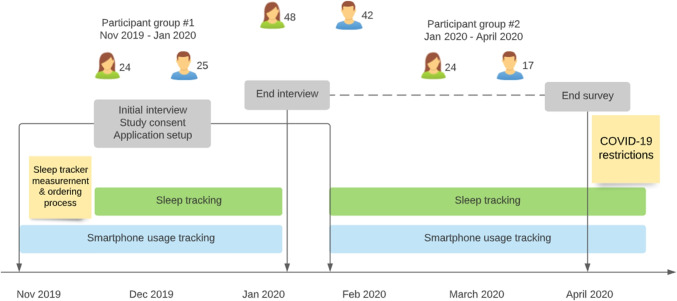
Overview of the study design and how the study was conducted. Two participant groups each lasted for approximately 3 months, after which the tracking applications were uninstalled and the data was collected from participants’ devices, as well as the cloud services of both AWARE framework (smartphone usage tracking) and Oura cloud (sleep tracking)

As seen in Fig. 2, we designed a study plan aimed at collecting information about the study participants’ sleep and smartphone use. Our study plan consists of 100 participants recruited in two batches of 50 participants each. We recruited participants through mailing lists and word of mouth. The participants received no monetary awards or other compensations for participation. Participants received a sleep tracking ring to be used during the study period and an Android smartphone if they did not possess one or have a model incompatible with the used tracking applications.

Our initial call attracted 452 potential participants. We invited two batches of 50 participants to the study and balanced the participant pool based on age and gender. We sent out invitations for the selected participants to meet with the researchers, consent to the study procedures, install the required logging applications, and measure their finger size for the ring. The delivery of the rings took approximately 2 weeks, after which the participants met with the researchers for a second time, received the ring, and any emerged problems with the logging applications were resolved. The first round of participants took part in the study between November 2019 and January 2020, and the second round of participants between February 2020 and April 2020.

In the initial meeting, the participants were informed of their consent and signed a consent form authenticated by the ethical board of the host university. Participants were given a participant ID that they used as members of the study to help anonymize their data. The participants received written and oral instructions on installing the tracking applications. Each participant’s collected data can be remotely monitored to capture any issues in the data collection.

Ninety participants responded to our invitations. Out of these 90, two participants dropped out mid-study; one participant had a skin condition that prevented wearing the wearable. One participant did not want to borrow an Android device for the study. Forty-eight (48) of the participants (53.3%) identified as female, 42 (46.6%) as male, the mean age of participants was 27 years (median of 24 years), with a standard deviation of 7.61 years, minimum age of 18, and maximum age of 61 years.

### Sleep data collection

The wearable ring consists of an accelerometer, temperature sensor, and an HR sensor that can measure daily activity and performance, as well as detailed sleep-related metrics, e.g., amount of deep and REM sleep, sleep disturbances, and sleep latency and efficiency. Table 1 shows a summary of the sleep-related metrics collected through the wearable ring.

**Table 1:** Sleep metrics provided by the wearable ring, through the vendors’ cloud service

Metric	Description
Total time in bed	Total time (in seconds) in bed, from going to bed to getting up
Total time of sleep	Total duration (in seconds) of sleep
Sleep latency	Time (in seconds) it took to fall asleep on the onset of bedtime
Sleep efficiency	Time (in percentages) spent asleep during bedtime
Light sleep	Amount of light sleep registered during the sleep period (in seconds)
Deep sleep	Amount of deep sleep registered during the sleep period (in seconds)
REM sleep	Amount of REM sleep registered during sleep period (in seconds)
Awake	Time spent awake after going to bed until get up in the morning
Total amount of sleep	Total sleep = REM + light sleep + deep sleep
RMSSD	Average heart rate variability (HRV) [99]
Average heart rate	The average heart rate registered during the sleep period
Restfulness	Number of sleep disturbances and restless events during the night
Sleep score	Aggregated sum of sleep rates variables to a score (1–100)

The accuracy of the tracking of sleep phases is closely related to analysis from polysomnography devices [63]. Participants were asked to join the ring vendor’s cloud service where their data are synchronized anonymously (participants used a pre-generated email to create their accounts). The participants’ data can then be accessed remotely and downloaded for further analysis.

The ring has an average battery life of 3–5 days and thus enables continuous tracking. The participant was asked to wear the ring as it best suits their everyday life. Some participants reported that they did not always wear the ring during the day because of their sports activities. They did not always wear it during the night — primarily due to simply forgetting about the ring. Typically the gaps in our sleep dataset last for 1–2 nights.

Two users had personal issues regarding sharing their sleep data for research and did not opt-in to share their sleep data through the wearable vendors’ cloud service. Eighty-six participants provided their sleep data on a total of 3990 unique nights. Participants shared an average of 43.76 nights with a standard deviation of 10.5. The largest sample comes from a participant who shared 68 nights’ sleep data.

We preprocessed the data to remove any abnormal or outlier nights when the participant’s nightly heart rate did not drop to the appropriate resting level due to, e.g., sickness or excessive alcohol intake. We used a threshold of 1.2$\times$ participants’ median nightly heart rate as a threshold, as advised by one of the co-authors, an expert in sleep research. We removed 152 nights of sleep data as outliers. These nights are already excluded from previously reported numbers.

### AWARE data collection

Tracking smartphone use and specific applications have been a valuable asset and methodology for researchers. Simple measurement of time spent using smartphones has been leveraged in research investigating human behavior [100] and emotions [101], or impact of a smartphone (over) use on sleep, and mental well-being [73].

Smartphone use is collected through the AWARE framework.^3^ AWARE is a toolkit that is available as a regular Android mobile application (client) or bundles in existing applications as a library. The client allows participants to “join” studies, where each participants’ data are tagged with their device ID as an identifier and automatically uploaded to a remote server. AWARE ensures continuous tracking of each enabled sensor so data gaps are minimized. AWARE is a widely used mobile sensing framework in smartphone-related studies and has proved to be a reliable method for collecting sensing data from participants’ personal devices [102].

#### Collecting data on application use

The AWARE “applications” sensor collects foreground applications from the participant’s smartphone. Foreground application is the visible application on the user interface of the smartphone. AWARE stores the timestamp and the application package name alongside the device identifier whenever the foreground application changes. We collected 1,007,964 foreground application change events from 79 unique participants after ensuring the collected data was intact.

The application’s sensors require specific system access on the participant’s smartphone called the accessibility option. Each android vendor has its own operating system, which can cause system options like accessibility or battery management options to behave differently. We have recognized occasional breaks in data collection due to the accessibility option being reset or the participant’s smartphone taking an excessively aggressive battery management stance, causing the background logging to cease^4^ momentarily. We adjusted any further analysis according to this by recognizing potential gaps in the data and only considering days when no significant gaps in data collection were identified.

## Analysis and results

This section introduces the smartphone use and sleep datasets and the pre-processing steps used to transform the application data to a more understandable and analyzable format. Then, we present the analysis methods and results to answer our research questions and initial hypotheses (see Section 3).

### Pre-processing methods

#### Sleep dataset and personal sleep quality

Definition of sleep quality differs for various individuals; 6 h of sleep might be good for a person and be bad or usual for another person. The wearable Oura ring presents the user with a sleep score (0–100) calculated according to a proprietary algorithm. Over time, the sleep score adapts and considers individual differences. Thus, the sleep score is not directly generalizable between participants. However, the score guidelines indicate a sleep score of 69 or less as non-optimal sleep, from 70 to 84 as normal sleep, and 85 or higher as optimal sleep quality.

**Fig. 3:**
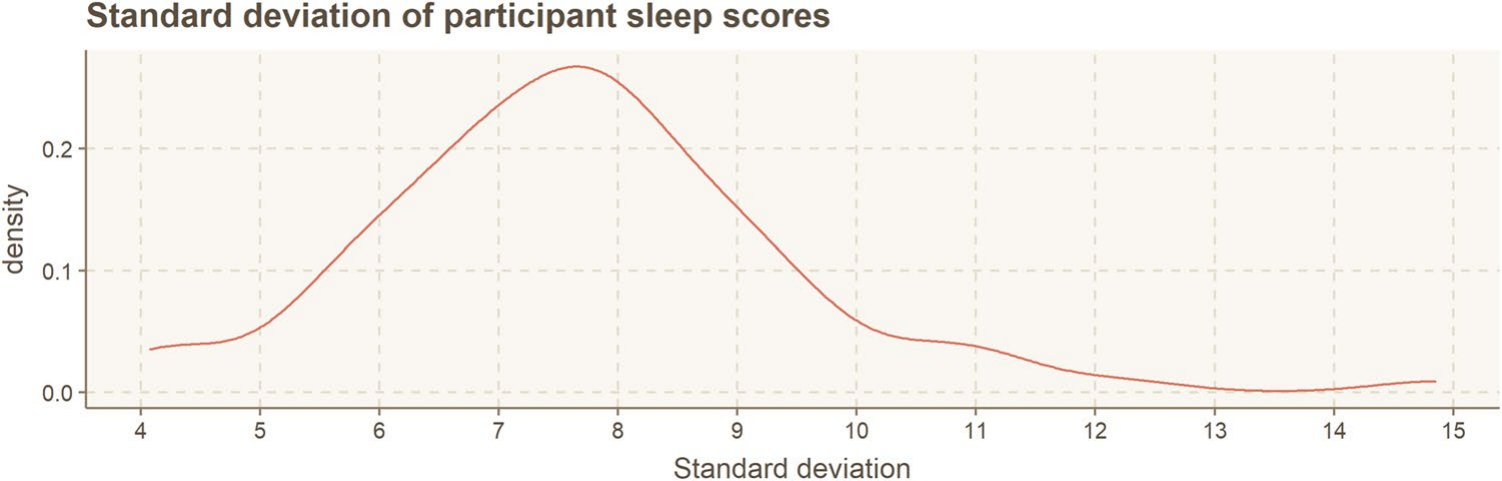
Summary of sleep score standard deviation, calculated for each study participant individually

Figure 3 shows a summary of the standard deviation in sleep scores within our dataset. The median sleep score is 74, with an interquartile range (IQR) of 69.9–78.3. Our co-author, a senior sleep researcher, motivated us to use a 5-point difference in sleep quality as a rule of thumb to differentiate between typical sleep quality and noticeably better or worse sleep quality. The empirical test rule specifies that 68% of samples fall within one standard deviation of the mean. However, for categorization based analysis, this creates a very biased dataset, with 68% of samples belonging to the average group and only 16% ($\times$2) of the samples in each fringe group. The standard deviation in our dataset is typically over 5 (*M* = 7.69). However, the 5-point rule as threshold creates more balanced categories, which are still based on the actual standard deviation, and the typical diversity of the IQR (78.3–69.9 = 8.4 points) in our dataset. As seen on the categorization list below, this process created an equal balance of categories.

We categorize sleep quality for each logged night into the following three categories:

- **Worse than usual (22.3% of samples)**: If the sleep score is *5 points lower* compared to the participant’s average sleep score, the sleep quality for that night is considered worse than usual.
- **Better than usual (25.4% of samples)**: Similarly, if the sleep score is *5 points higher* than the average sleep score, the sleep quality is considered better than usual.
- **Usual (52.3% of samples)**: Lastly, any sleep score that falls within 5 points of the average sleep score is considered normal.

We also removed outlier nights from the dataset, including, e.g., the night wherein the people had abnormally high heart rate indicating sickness or excessive alcohol consumption, to not skew what we would consider “normal” behavior in our analysis. How people act in these more extreme scenarios is beyond the scope of this paper.

#### Smartphone use dataset

We cross-referenced the Google Play application store to find out each applications’ application category using the application package name. Examples of Google Play categories are, e.g., social media, entertainment, productivity, and different types of games. Since 2019, the Google Play Store has dropped the term “game” from the game categories; thus, applications in a category such as “Simulation” consist of *simulation games*.

We also computed the duration of each *application use session* by measuring the time between a foreground change to application A and the following application B — resulting in-use time for application A. Any outliers caused by potential logging errors (application B missing) and simple outlier behavior (e.g., using an application for several hours) were removed according to a threshold set by mean use time plus one standard deviation. A total of 541 outlier use sessions (0.05% out of the original 1.007M) were discarded, resulting in 1,007,423 application use sessions to be analyzed.

##### Typing applications

Whenever the Android OS shows a visible keyboard to the user, it is considered a unique application that is (i) shown in the *foreground* and (ii) logged by AWARE as an individual application. We manually searched our dataset for all package names that are related to keyboard or typing, e.g., the standard Android keyboard *Gboard* has a package name of “com.google.android.inputmethod.latin.” We then assign each of these keyboard applications the “Typing” application category. This category also includes third-party keyboards, e.g., Microsoft SwiftKey.

For our analysis, we consider these smartphone use metrics in the following section(s):

- **Smartphone use:** Overall sum (in seconds) of using *any* smartphone application.
- **Application category use:** Combined sum (in seconds) of smartphone application use of applications that belong in the same Play Store *category*.
- **Typing:** Combined sum (in seconds) of having the smartphone keyboard visible to the user; i.e., use of smartphone applications in the “*Typing*” category.

#### Merging sleep dataset with smartphone use dataset

We merged the sleep dataset with the smartphone use dataset based on the participant ID and the date. We ended up with 75 users’ data after merging the two datasets. To investigate the effect of using smartphones on sleep quality throughout the day, we divided the merged dataset into several time windows: smartphone use in bed, smartphone use in bed and 1 h ago, smartphone use in bed and 2 h ago, etc. up to 16 h before going to bed (with 1, 2, 4, 8, 16 intervals). The sleep tracking ring can identify (i) the moment the user falls asleep and (ii) the moment the user lies down, as humans’ heart rate significantly changes when we are standing, sitting, or lying down. This allows us to measure the time spent in bed while awake (“in bed”). As we describe these periods, we include the bedtime in all annotations, e.g., “one hour ago” includes the hour before bedtime *and* the bedtime.

**Fig. 4:**
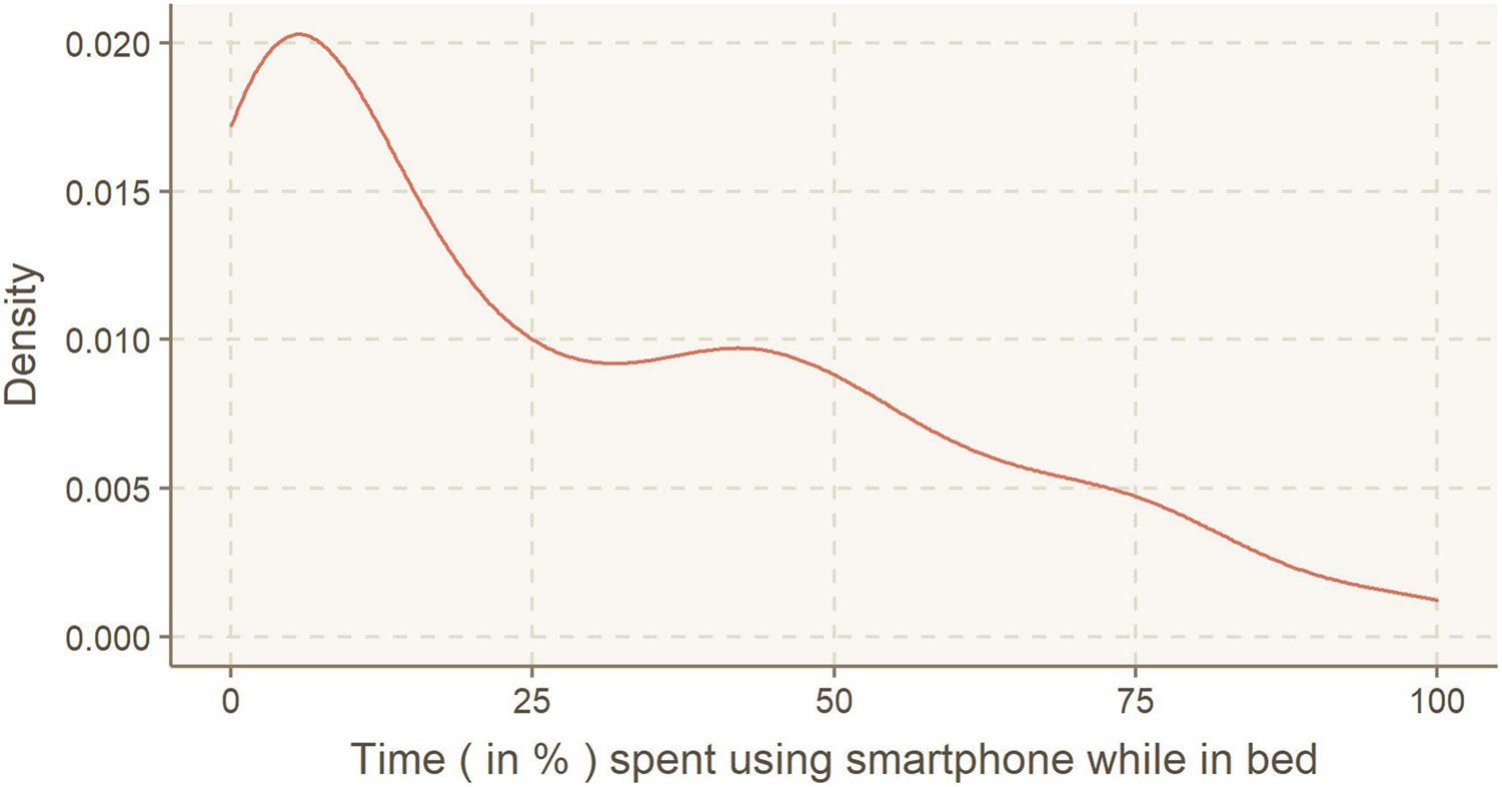
Distribution of smartphone use while in bed, presented as % of time spent in bed

The participant pool had an average sleep latency of 723.7 s (12 min and 3.7 s), with an IQR of 300–960 s. On average, the participants used their smartphones in bed for 322.8 s (5 min and 22.8 s), with an IQR of 43.7–456. Participants spent an average of 42% of their time in bed using their smartphones, with an IQR of 5.87–55.5% (Fig. 4).

### Analysis

We first analyze how smartphone use affects sleep quality in general, then how the use of distinct application categories, and lastly, how more active and engaging smartphone use in the form of smartphone use that requires *typing* influences sleep quality.

We compare users’ behavior between use in-bed and outside of bed their sleep quality according to time intervals starting from 1 h before bed, 2 h before bed, etc. up to 16 h before bedtime. We wish to understand better if smartphone use has any specific prolonged effects on sleep quality or if any observable effects are quickly “forgotten” before sleep. Sleep hygiene guidelines often cite the importance of a “winding down” period before and during bedtime. We wish to see if any observable differences exist on the impact on sleep quality when analyzing smartphone use in bed versus smartphone use 1 h before bedtime.

#### Generic smartphone use and sleep quality

**Fig. 5:**
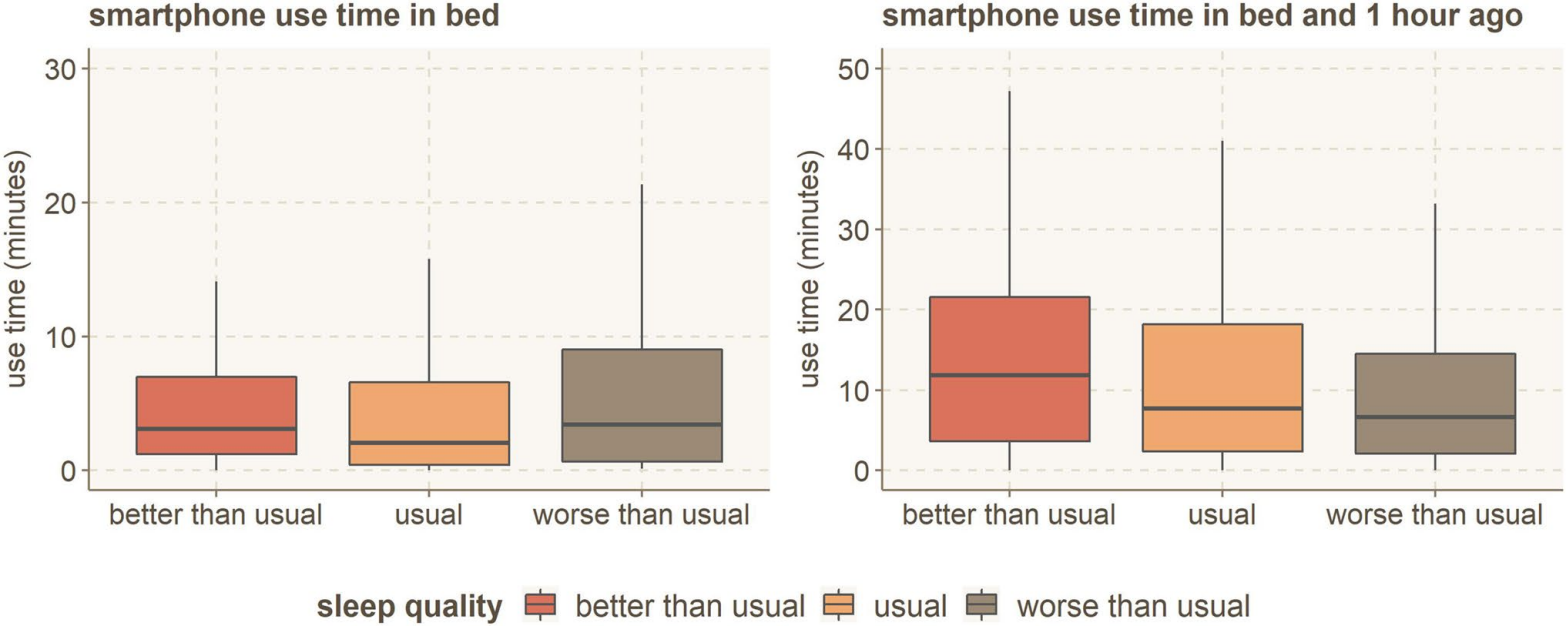
Average smartphone use time, grouped by individual’s sleep quality while in bed, and starting from 1 h before bedtime (including bedtime)

First, we aim to answer **RQ1; whether smartphone use, in general, can be shown to have an impact on sleep quality.** To investigate the general smartphone use at different times, we summed all the application usage from of each participant, for each day, at specified time windows (of 1, 2, 4, 8, and 16 h). We then grouped the resulting data for nights with similar sleep quality (worse than usual, usual, better than usual). The average smartphone use time *in bed* and *1 h ago* with different sleep quality is shown in Fig. 5. More *in-bed* smartphone use frequently leads to worse sleep quality and vice versa. The behavior is similar for all out-of-bed smartphone usage time windows (1h, 2h, 4h, etc.).

**Table 2:** One-way ANOVA statistical test results to investigate the effect of smartphone use on sleep quality

Starting point of time window	*F*-value	*P*-value
Smartphone use *in bed*	2.263	.106
Smartphone use starting from *1 hour* before bedtime	5.587	<.005**
... *2 hours* before bedtime	8.118	<.005**
... *4 hours* before bedtime	4.812	<.01**
... *8 hours* before bedtime	4.082	.02*
... *16 hours* before bedtime	2.248	.106

*p*-value * <0.05; *p*-value **< 0.01

We used the one-way ANOVA statistical test to verify the significance of the association between the average smartphone use time and sleep quality categories. The results are shown in Table 2. To summarize the findings on general smartphone use and sleep quality, overall smartphone use can influence how well you sleep for the latter part of the day (8 h or less before sleep). Still, there is no significant difference in sleep quality regardless of how much one uses their smartphone while in bed or through the whole 16-h day. Prioritizing one’s smartphone use on the after-work hours — the up-to 8-h period before bedtime — can lead to improved sleep quality. Also, as can be seen in Fig. 5, the people who use smartphones more **in bed** have **worse than usual** sleep quality. While this is not statistically significant (*p* = .106), it indicates that smartphone use in bed could harm sleep quality and should be further investigated.

**Fig. 6:**
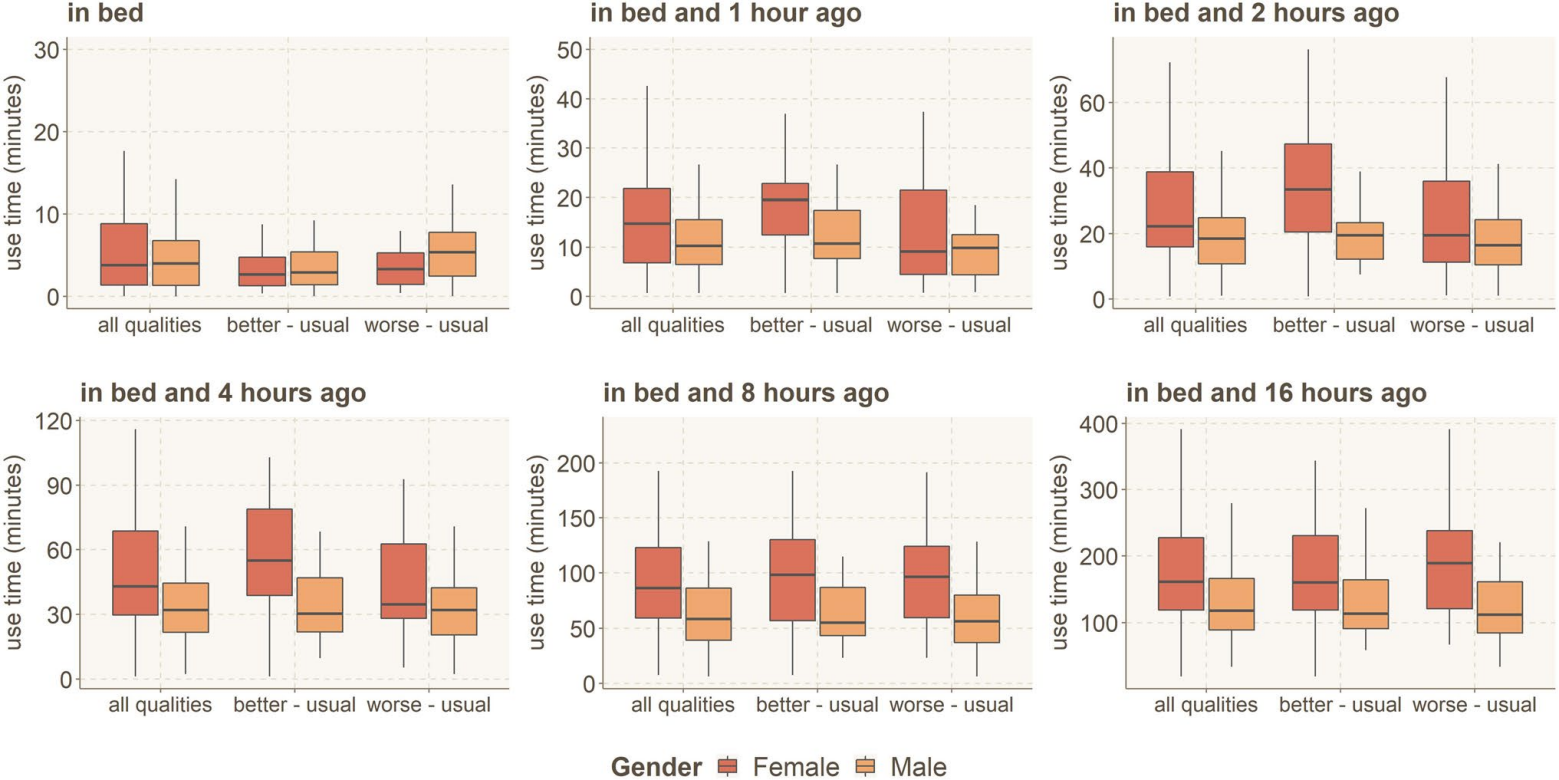
Average smartphone use time based on gender and different sleep quality, usual = use time when sleep quality is in usual range (±5), better = use time when sleep quality is greater than the usual range, worse = use time when sleep quality is less than the usual range. *Y*-scale shows the difference in use time for nights with, e.g., better than usual sleep (“better - usual”)

Next, we investigate whether gender impacts the relationship between smartphone use and sleep quality. For each participant, we calculated the difference of average smartphone use time between the nights with worse than usual and usual sleep quality and the nights with better than usual and usual sleep quality. The results between groups are shown in Fig. 6 for all the time windows. In all the time windows, females’ usage time is higher. We used the one-way ANOVA statistical test to verify the significance of the association between the average smartphone use time and the participants’ gender. The results are shown in Table 3.

As shown in Fig. 6, only in *bedtime* the males slept worse than usual when they used smartphone more. In the other time windows, the females slept worse than usual when using smartphone more than usual. We used the one-way ANOVA statistical test to verify the significance of the association between the difference of average smartphone use time for participants with better than usual and worse than usual from the ones who had usual sleep quality and participants’ gender. The results are shown in Table 4.

**Table 3:** One-way ANOVA statistical test results to investigate the effect of gender on smartphone use

Starting point of time window	*F*-value	*P*-value
Smartphone use *in bed*	3.726	.708
Smartphone use starting from *1 hour* before bedtime	10.15	.001***
... *2 hours* before bedtime	13.61	.0002***
... *4 hours* before bedtime	21.96	5.32e-06***
... *8 hours* before bedtime	27.52	4.14e-07***
... *16 hours* before bedtime	25.53	1.02e-06 ***

*p*-value *<.05; *p*-value **<.01; *p*-value ***<.001

**Table 4:** One-way ANOVA statistical test results to investigate the effect of gender on smartphone use and sleep quality

Starting point of time window	*F*-value	*P*-value
Smartphone use *in bed*	0.982	.325
Smartphone use starting from *1 hour* before bedtime	3.89	.051*
... *2 hours* before bedtime	8.972	.003**
... *4 hours* before bedtime	4.812	.008**
... *8 hours* before bedtime	0.0867	.354
... *16 hours* before bedtime	8.167	.005**

*p*-value *<.05; *p*-value **<.01

#### Application categories and sleep quality

Next, we focus on analysis on **RQ2 — Do specific application types cause an influence on sleep quality? — and RQ3 — Does typing-related activity influence sleep quality?**

**Fig. 7:**
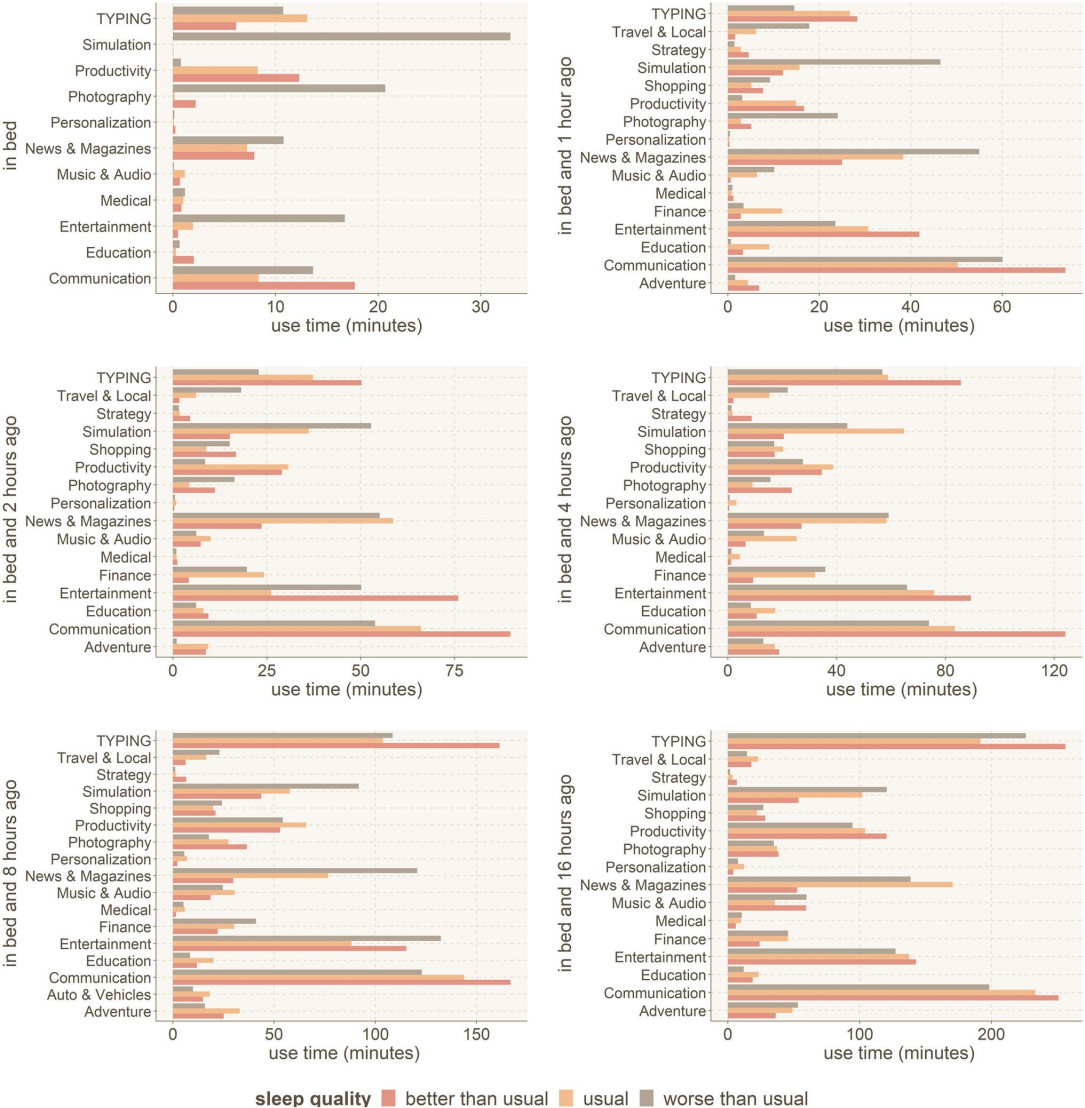
Use time for each applications’ category in different time windows, and before nights with better/usual/worse than usual sleep quality. Results indicate longer usage times leading to differences in sleep quality

To investigate the impact of application categories on sleep quality, we calculated the total use time of each application that participants used before each night and then grouped the results according to the sleep quality categories. We used the one-way ANOVA statistical test to compare the sleep quality category and total use time in each application category. The results of the statistical test for each category at different times of the day are shown in Table 5. We have omitted results with *p*-value > .25 from the table. We have adjusted for multiple test comparisons with the Bonferroni correction and noted the results with the typically significant result (*p* < .05) with * and Bonferroni corrected results (*N* = 6, *p* < .01) with **. Not all types of applications were observed in all time windows in our dataset, explaining some application types being omitted in these results and following results, e.g., in Fig. 7.

**Table 5:** One-way ANOVA statistical test results to investigate the effect of use time of different application categories on sleep quality

Time window	Category	*p*-value	Category	*p*-value
In bed	Entertainment	6.42e-07 **	Simulation	.000286 **
	Photography	.248		
One hour	Photography	.00884 **	News and magazines	.106
	Communication	.194		
Two hours	Typing	.0956	Finance	.0614
	Medical	.0459 *	Communication	.133
	Personalization	.238	Photography	.102
	Adventure	.187		
Four hours	Typing	.00519 **	Travel and local	.00332 **
	Communication	.0134 *	Finance	.0254 *
	Medical	.0103 *	Photography	.0878
	Education	.162	Personalization	.198
	Shopping	.242	Strategy	.151
Eight hours	Typing	.0011 **	Travel and local	.0355 *
	Simulation	.0466 *	Finance	.0662
	Medical	.0617	Adventure	.0862
	Communication	.22	Strategy	.167
16 hours	Typing	.00807 **	Finance	.0657
	Travel and local	.0867	Communication	.177
	News and magazine	.231	Music and audio	.131
	Entertainment	.221	Simulation	.252
	Shopping	.164		

*p*-value *<0.05 for generic significance and *p*-value **<0.01 for Bonferroni corrected values

In addition to looking at statistical differences and the effect size from the statistical tests, we also want to look at any substantial differences between application use that result in different sleep quality. We average the daily use of each application category and group them according to the sleep quality categories during the following night. Results of each application category that was observed in each of the time windows are shown in Fig. 7.

For the categories with statistical significance, the difference is clear between better and worse than usual sleep quality in Fig. 7. For example, use of **Entertainment** and **Simulation** (games) applications while in bed tends to cause sleep quality to decline. Use of **Travel** applications *after* work and *before* the late evening can lead to worse sleep quality, while **Typing** at that time can improve sleep quality. This result highlights the importance of the winding down period before sleep, its requirement specifically while in bed.

The visualization in Fig. 7 also highlights **potential** application categories which could have an impact on sleep quality, in addition to those shown to be of statistical significance:

- **In bed:** Use of **Photography** and **Typing** applications in bed can lead to *worse than usual* sleep quality, while **Productivity** and **Communication** can lead to *improvement* in sleep quality.
- **Late evening (1 to 2 h before bed):** **Simulation** and **News** can lead to *worse* sleep quality, while **Typing**, **Productivity**, **Entertainment**, and **Communication** could *improve* sleep quality.
- **After-work (up to 4–8 h before bed):** Use of **News** and **Finance** applications typically can lead to *worse* sleep quality, while **Productivity**, **Entertainment**, and **Communication** could *improve* sleep quality.

The largest shifts between use in bed and outside of bed appear to be for **Typing** and **Entertainment** applications. Both show that use of such applications while *in bed* leads to *worse* sleep quality, but out of bed use is associated with *improvements* in sleep quality. Use of both **Productivity** and **Communication** applications tend to be always associated with *better* sleep quality.

### Effect of smartphone use in bed to detailed sleep metrics

**Fig. 8:**
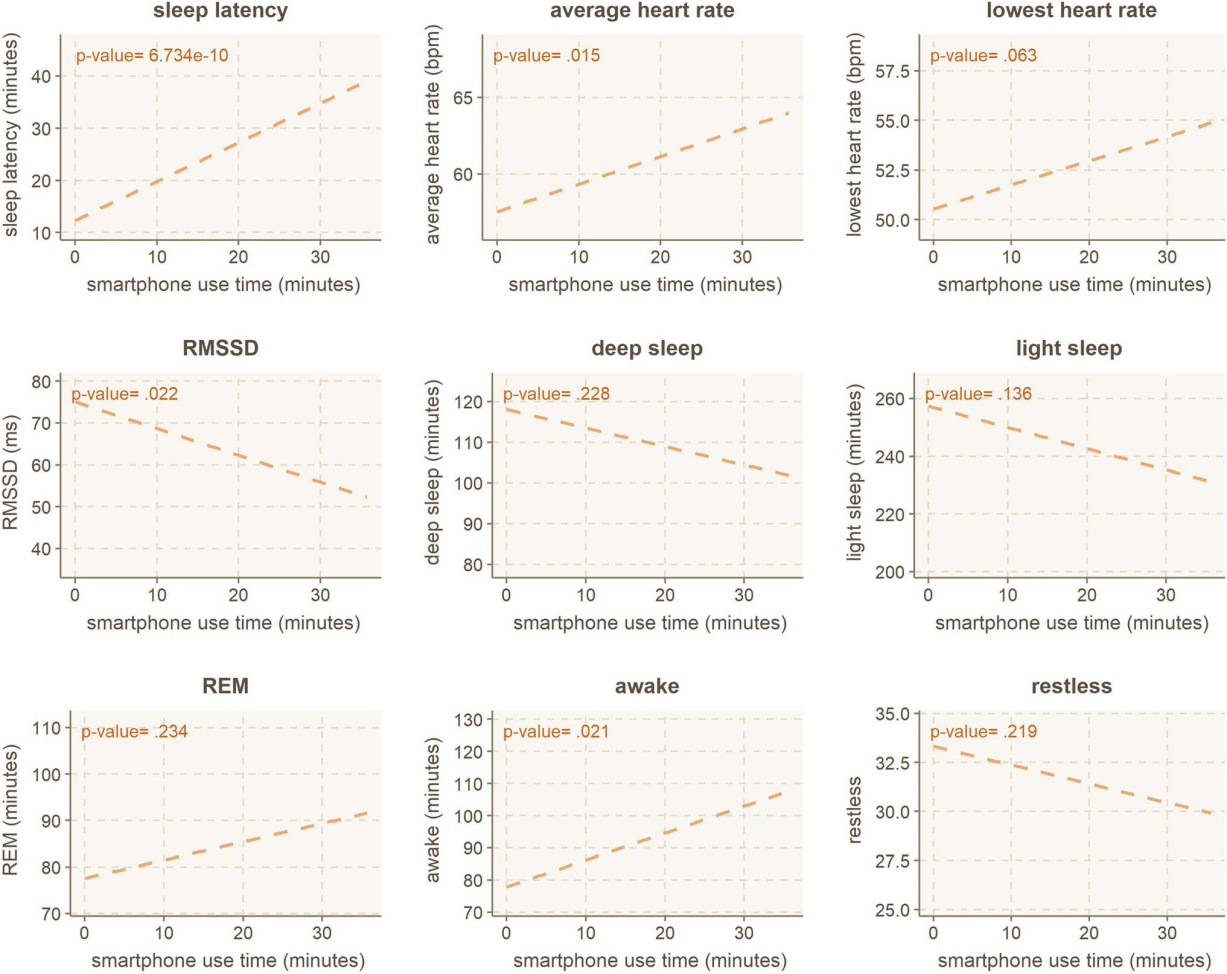
Linear regression model for smartphone use *in bed* and detailed sleep metrics

Finally, we delve into our last **RQ4 — investigating whether in our dataset we can observe the influence of smartphone use on the** *detailed* **sleep metrics.** The wearable ring offers more detailed sleep metrics than a generic sleep score. The details of the metrics are listed previously in Table 1 and include metrics like for example, sleep latency (the time it takes to fall asleep), time spent in different sleep phases (light, deep, REM), or disruptions to your sleep. We look at the correlation of using *smartphones while in bed* on each of the nine metrics using standard linear regression. Four out of the nine correlations are statistically significant (*p* < .05 for sleep latency, average heart rate, heart rate variability, and awake time), with the lowest heart rate being borderline (*p* = .063). The linear regression and corresponding *p*-values are presented in Fig. 8.

Investigating the results from Fig. 8 in more detail, we can observe that increased smartphone use causes a decline in seven out of nine variables. Two remaining variables are increased duration of REM sleep and decrease in restless periods during the night (both *p* > .05). Next, we will briefly go through the individual effects on sleep metrics.

#### Sleep latency and being awake

Use of smartphones in bed has a statistically significant effect on both sleep latency and the duration of awake phases during the night (including bedtime). While it is logical that *one cannot fall asleep while using their smartphone*, or at least, is very unlikely to do so, the use of smartphones can significantly *delay falling asleep* and *cause awake periods later on*. From observing the intersection between use time and sleep latency, no smartphone use (*x* = 0 min) correlates to roughly 12 min of sleep latency (*y* = 12 min), while 20 min of smartphone use (*x*-axis) correlates to 28 min (*y*-axis) of sleep latency. Roughly speaking, every 5 min you spend on your smartphone while in bed, your sleep is delayed by 4 min. Similarly, every 10 min of smartphone use in bed causes the awake time to increase by 9 min.

#### Heart-rate variability

Night-time heart rate is an indicator of how well you have rested during the night. Similarly, heart rate variability indicates how well you have recovered and how well your body is, e.g., coping with stress. Higher heart rate variability indicates *better* recovery capabilities. Thus, all HR-related variables negatively impact smartphone use in bed.

#### Sleep phases

The effect on sleep phases (deep, light, REM) is non-significant yet relatively strong, with an example of 30 min smartphone use period while in bed leading to 20 min decline in deep sleep and 30 min decline in light sleep. In comparison, REM sleep duration only increases by 10 min for use time of 30 min. Thus, a 30-min use session while in bed could lead to a compounding 1 h of sleep loss during the night.

#### Typing in bed

**Fig. 9:**
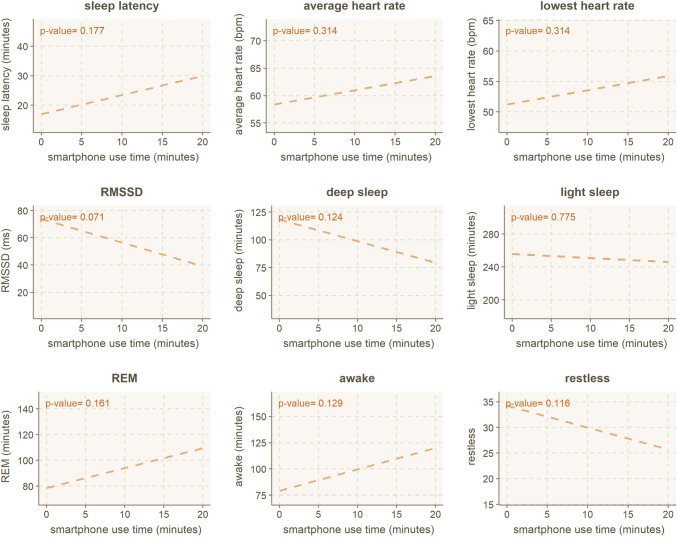
Linear regression model for typing in bed and detailed sleep metrics

Finally, we wanted to explore if active and engaging smartphone use — through applications requiring typing — would have similar or otherwise identifiable effects on the sleep metrics. Similarly to overall smartphone use, we use the standard linear regression analysis. Our findings do not indicate any statistically significant effects. However, the behavior seems similar to overall smartphone use in all nine variables. The results can be seen in Fig. 9. Intuitively, it seems that passive use of smartphones (consuming information) would have less impact on falling asleep and sleep quality than active and more engaging smartphone use (producing information, e.g., through typing), presenting a fascinating future research avenue.

## Discussion

Sleep plays a crucial role in the quality of human daily life [23], and sleep hygiene is an essential part of daily routines. Sleeping well and good sleep hygiene can improve brain functionality, memory, and cognitive capabilities [3]. Technology is integral in daily human life, and excessive use of technology can disrupt these daily activities. The impact of technologies and digital services on people’s mental, physical, and emotional health is defined as digital well-being. Digital well-being means comprehending and recognizing the positive and negative effects of digital services and being aware of ways to balance using technology and daily activity to improve digital well-being. Increased smartphone use in bed can potentially lead to a decrease in sleep quality, and this effect was mainly focused on specific aspects of sleep, e.g., sleep latency. Other studies highlight similar results. For example, electronic device use in bed reduces sleep duration and quality in adults [103], and in-bed electronic social media use has direct associations with insomnia, daytime sleepiness, mood, and sleep duration in adults [21, 22]. Both bedtime procrastination and poor self-regulation caused by smartphone addiction lead to poor sleep quality [104]. Reduced sleep quality is likely due to the effects on independent sleep-related variables such as sleep latency [17, 82] influence heart rate, awake time, and reduce heart rate variability (meaning increased stress levels). The overlying reason could be screen time [88], as smartphone screens emit the strong blue part of the visible spectrum of light [12–14]. This causes secretion inhibition of melatonin and decreases sleepiness by affecting the sleep-wake cycle [15, 83]. Smartphone use in bed tends to increase the time to fall asleep by the ratio of 80%, so not all time is effectively wasted, but it still postpones falling asleep by a significant amount. It also leads to increased periods of being awake during the night. Typical sleep health and sleep hygiene instructions call for a *winding down period* before bed, and if such period is neglected, it can likely lead to your mind still racing during the night and keeping you up at times. Similarly, smartphone use seems to (*p* > .05) increase the ratio of REM sleep to deep and light sleep, which could be attributed to increased brain activity before falling asleep. The things we think of or worry about before bedtime often re-emerge within our dreams.

The winding down periods or proposed winding down routines before bed typically rely on relaxing and *passive* activities, and as such, some smartphone use can be categorized as simply *consuming information*. Other activities might require a more active role from the user. To differentiate between passive and active actions on the smartphone, we looked into the user engaging with their device through typing activities. Typing activities on the smartphone while in bed do not seem to have any specific impact outside of general smartphone use (is not statistically significant). The cognitive functions required to “create” information (e.g., text) or engage in conversations do not impact sleep quality differently than the functions required to consume incoming information (e.g., video, images, text, or audio). However, as can be observed in Fig. 7, active use of smartphones through typing outside of bedtime (after work and before the late evening) has a strong positive effect on sleep quality. Females tend to use smartphones more than their male counterparts during all parts of the day. The communication application category tends to be the most used, and the work of Andone or Chen [105, 106] also concluded that females spend more time in communication and social apps. In comparison, males spend more time playing games. Altogether, studies revealed that overuse of technology can lead to poor sleep quality [1, 22, 73–77, 77–81], and intensity of physical health problems such as blurred vision, pain in the hand, back, neck, and head [70], e.g., migraine that can lead to sleep quality reduction and daytime sleepiness increment [71].

### How do specific application categories impact sleep?

Our findings showed that the use of simulation games generally tends to reduce sleep quality, perhaps due to the overload of certain cognitive functions and memory required to interact with such games, especially over the course of a whole day through a smartphone. This is also described by other investigators [107–109]. Miskoff et al. [110] illustrate that playing video games before bed does not have any effect on sleep quality. However, our findings show that playing strategy and adventure games in bed do reduce sleep quality. It would be better for health and well-being not to access games requiring high cognitive functions through a smartphone and to limit playing such games only at certain times of the day (instead of throughout the day). As investigators [111, 112] suggest, the use of entertainment applications in bed seems detrimental to sleep quality, and the use of such applications during the evening instead (up to 4 h *before* bedtime) could be beneficial. During bedtime, the males who sleep worse than usual also tend to use smartphones more than females. Other researchers note that games could be a reason for this, but only a handful of participants played games on their mobile devices in our sample. Looking at the results of Fig. 6, duration of smartphone use does not tend to have an impact on male sleep quality outside of when it is used in bed — and where excess smartphone use tends to lead to worse sleep quality, albeit by only a small margin.

We found that the cases where participants used Travel applications after work and before the late evening had worse sleep quality than usual. They might be on a personal or work trip. It seems that they were under stress and anxiety, got tired, or were sleeping in unfamiliar surroundings, and accordingly had low sleep quality. On the contrary, using the applications that need Typing at the time can improve sleep quality.

Our findings showed that social media or communication applications do not lead to decreased sleep quality. One explanation is that smartphone use, in general, leads to decreased sleep quality, not social media specifically. This result is somewhat contrary to other researchers’ results that showed using social media leads to decreased sleep quality [17, 94–98]. Alternate explanation for the cited results could be that in their case, just the overall use of smartphones was the culprit, not social media specifically. The cited works investigated the effect on adolescents and undergraduate students specifically. In these populations, the “I don’t want to go to bed yet” tendency could exist regardless of *what* activity keeps them awake. Smartphones, obviously, are a prime culprit for such an effect. Alternatively, since the cited studies were qualitative, not quantitative (none of the cited works had bio-statistical data like our study), participants may sometimes trick their minds about the adverse effects of social media on their sleep quality when answering the questionnaires.

Our potential results showed that the use of different applications throughout the day can indicate the user’s lifestyle, related to the type of work they are doing, and their health condition and daily activities. Whether the applications are used for personal reasons or if the use is work-related, they have a similar effect on sleep (see Fig. 7), and people increasingly work outside of their typical 9-to-5 work hours. Particularly, the use of News applications throughout the day seems to lead to a decline in sleep quality. As our study period overlapped with the initial stages of the COVID-19 pandemic (from December 2019 to April 2020), being overwhelmed by the news at this time could explain a distraught that led to worse sleep quality. The people who used finance applications from 2 to 16 h before going to bed had a low sleep quality. Perhaps they work at banks or financial companies, and their job is stressful, or they might have personal financial issues and think about financial problems more than the others, which leads to stress and anxiety.

### Takeaways and recommendations

The results of this study support existing evidence and literature through a novel data-driven approach that logs both participant sleep quality metrics and analyzes those metrics alongside quantified smartphone usage metrics. To summarize our findings in the context of our research questions (RQ1–RQ4), our first goal was to investigate *RQ1: Does more smartphone use during bedtime or throughout the day generally translate into worse or better sleep quality?* The first hypothesis (H1) was **true**; use of smartphones during bedtime particularly decreases sleep quality and has a provable impact on specific metrics of sleep quality. The second research question looked into the use of *specific* applications: *RQ2: Does the use of specific application types correspond with worse or better sleep quality?* As can be observed in Table 5 and Fig. 7, certain application categories were used differently across different sleep quality categories (better than usual, usual, worse than usual). The answer to RQ2 is **yes**, using applications such as Entertainment and Simulation (games) in bed can reduce sleep quality. In addition, using Travel applications before late evening can decrease sleep quality, while using applications that need typing at that time can improve sleep quality. For the third question on differences between active and passive smartphone use, *RQ3: Does the use of applications requiring active involvement in the form of typing have an impact on sleep quality?*, Use of applications that need active engagement like typing after work and before the late evening was associated with better sleep quality. This is an interesting finding that warrants further research. Lastly, active smartphone use in the form of *typing* did not reveal any significantly differing results from generic smartphone use, which was investigated under *RQ4: How do smartphone use and typing activities influence detailed aspects of sleep (e.g., sleep phases, sleep latency).*

Given our findings, our most important recommendation is to leave your smartphone out of your bed and also out of your bedroom. One should use an alarm clock instead of using the smartphone’s alarm. If the smartphone must be used as an alarm clock, e.g., while travelling, setting it in airplane or do not disturb mode to reduce disruptions and placing it out of reach is advised. To help the users be focused and less harmed by smartphones, application developers should design more strict and severe limitations to smartphone use time and even suggest limiting the use of specific applications. Therefore, researchers need to investigate the effect of limiting smartphone use and specific applications on sleep quality and overall well-being.

Second, while previous research has shown links of, e.g., social media use decreasing sleep quality, our data did not support this. Yet, in addition to limiting smartphone use in bed, we do encourage people to pay attention to how different applications affect their mental states. We acknowledge a potential issue with our sample size in this study and advocate for more research on the effects of specific applications or application categories on sleep.

### Limitations

Our study indicated that the use of smartphones during bed-time might notably decrease sleep quality and have a provable impact on specific metrics of sleep quality such as sleep latency, awake time, average heart rate, and heart rate variability. However, we are, for now, uncertain which one is the cause and which is the effect. Does the increase in smartphone use in bed lead to poor sleep quality? Or does poor sleep quality lead to an increase in smartphone use? In order to get generalized results, more data should be collected from a wider variety of study subjects.

## Conclusion

This paper collected and investigated a 4-month-long dataset of sleep data from a sleep tracking device (Oura ring) and smartphone use data collected from the participants’ personal devices. We analyze a dataset from 75 study participants and reveal novel findings of the relationship between smartphone use and sleep quality. Given the increasing use of smartphones and their impact on people’s mental, physical, and emotional health, we need to find methods to improve digital well-being.

Our findings indicate that using smartphones in bed leads to decreased sleep quality. Therefore as a healthy guideline, try to refrain from using technology or entertainment sources once you go to bed and during your bedtime. Instead, let your brain process the information from such sources before bedtime. This part of the evening should function as a pre-winding down period.

As our dataset had access to more fine-grained sleep metrics than commonly used (e.g., self-reported sleep/wake times or self-assessment surveys), we managed to uncover and quantify the effect of technology use on specific sleep quality metrics, e.g., sleep latency, heart rate, and sleep stages. Our results show that using smartphones in bed leads to increased sleep latency, higher average heart rate, and lower heart rate variability — which are directly related to the quality of rest and are indicative of *worse* rest quality. Smartphone use can also lead to a higher proportion of awake periods during the night, reducing total sleep time.

Our results strongly highlighted the previous health guidelines of winding down before bed to improve sleep quality and show future directions for improving one’s sleep health and sleep hygiene.

## Footnotes

1 https://www.bankmycell.com/blog/how-many-phones-are-in-the-world

2 https://www.oberlo.com/statistics/how-many-people-use-social-media

3 https://awareframework.com/

4 https://developer.android.com/training/monitoring-device-state/doze-standby
